# Improved Yield and Photosynthate Partitioning in AVP1 Expressing Wheat (*Triticum aestivum*) Plants

**DOI:** 10.3389/fpls.2020.00273

**Published:** 2020-03-17

**Authors:** Kamesh C. Regmi, Kalenahalli Yogendra, Júlia Gomes Farias, Lin Li, Raju Kandel, Umesh P. Yadav, Shengbo Sha, Christine Trittermann, Laura Short, Jessey George, John Evers, Darren Plett, Brian G. Ayre, Stuart John Roy, Roberto A. Gaxiola

**Affiliations:** ^1^School of Life Sciences, Arizona State University, Tempe, AZ, United States; ^2^Australian Centre for Plant Functional Genomics, The University of Adelaide, Adelaide, SA, Australia; ^3^Department of Biological Sciences, BioDiscovery Institute, University of North Texas, Denton, TX, United States

**Keywords:** wheat (*Triticum aestivum*), H^+^-PPase (proton pyrophosphatase), reduced carbon partition, yield, PPi metabolism

## Abstract

A fundamental factor to improve crop productivity involves the optimization of reduced carbon translocation from source to sink tissues. Here, we present data consistent with the positive effect that the expression of the *Arabidopsis thaliana* H^+^-PPase (*AVP1*) has on reduced carbon partitioning and yield increases in wheat. Immunohistochemical localization of H^+^-PPases (TaVP) in spring wheat Bobwhite L. revealed the presence of this conserved enzyme in wheat vasculature and sink tissues. Of note, immunogold imaging showed a plasma membrane localization of TaVP in sieve element- companion cell complexes of Bobwhite source leaves. These data together with the distribution patterns of a fluorescent tracer and [U^14^C]-sucrose are consistent with an apoplasmic phloem-loading model in wheat. Interestingly, ^14^C-labeling experiments provided evidence for enhanced carbon partitioning between shoots and roots, and between flag leaves and milk stage kernels in AVP1 expressing Bobwhite lines. In keeping, there is a significant yield improvement triggered by the expression of *AVP1* in these lines. Green house and field grown transgenic wheat expressing *AVP1* also produced higher grain yield and number of seeds per plant, and exhibited an increase in root biomass when compared to null segregants. Another agriculturally desirable phenotype showed by AVP1 Bobwhite plants is a robust establishment of seedlings.

## Introduction

According to the Food and Agriculture Organization of the United Nations, wheat (*Triticum aestivum* L.) is the largest primary commodity in the world, cultivated on more land area than any other commercial crop (∼220 Mha) with global production of over 700 million tons, a total global annual export value of close to US$50 billion, and accounts for a fifth of our total available dietary calories^1^. Given that plant productivity is dependent on how organic carbon and other nutrients are acquired and partitioned (Khadilkar et al., 2015), it is imperative that we mechanistically dissect this complex process in important crops like wheat, and identify rate-limiting steps to improve yield (Ainsworth and Bush, 2010; Braun et al., 2013; Dasgupta et al., 2014; Yadav et al., 2015).

The synthesis of primary photosynthate, sucrose (Suc), in wheat starts with carbon fixation into triose phosphates in the photoautotrophic mesophyll cells in source leaves. It is then either transiently stored in chloroplasts and vacuoles, or is transported via the phloem to heterotrophic sink tissues and/or used as the substrate for starch reserves in the grain endosperm (Trethewey and Smith, 2000; Bahaji et al., 2014; Griffiths et al., 2016; Kumar et al., 2018). Wheat leaf blades have the characteristic parallel venation pattern of a monocot, with intermediate and small sized veins, together called the minor veins, constituting the collection phloem that channel into the major veins or the transport phloem (Fritz et al., 1983; Russell and Evert, 1985). Transfer of the photoassimilate from the collection phloem to the transport phloem occurs via the transverse veins that connect adjacent parallel veins (Kuo et al., 1972). None of the monocots studied thus far have intermediary cells in the phloem that are typical of plants that employ the polymer trapping mechanism (Esau, 1969; Rennie and Turgeon, 2009; Turgeon, 2010; Braun et al., 2013). Early anatomical (Kuo et al., 1974) and micro-autoradiography studies (Altus and Canny, 1982, 1985) suggested a passive symplasmic path for sucrose loading in wheat source leaves.

However, more recent studies implicate an active apoplasmic phloem loading strategy in wheat leaves. In a eudicot like arabidopsis, the role of Group 2 SUT proteins in apoplasmic loading is well-established (Truernit and Sauer, 1995; Stadler and Sauer, 1996; Bürkle et al., 1998; Wright et al., 2003; Srivastava et al., 2008; Ayre, 2011). Aoki et al. (2002) isolated three single-copy homeologous sucrose symporter genes *TaSUT1A*, *1B*, and *1D*, encoding 98% identical proteins, and later found that while the *TaSUT1* encoded transcripts were localized in the companion cells in source leaves, the *TaSUT1* epitopes localized to the plasma membrane of sieve elements in large, intermediate, and minor veins in source leaves (Aoki et al., 2004). Furthermore, upon feeding symplasmic tracer dye 6-Carboxyfluorescein diacetate, Aoki et al. (2002) also found that the sieve element companion cell (SE-CC) complexes of minor veins in source leaves were symplasmically isolated, suggesting a predominantly apoplasmic phloem loading strategy in wheat.

The consensus model for apoplasmic phloem loading in wheat would therefore hold that, sucrose first passively diffuses via plasmodesmata from the mesophyll cells (Giaquinta, 1983) to the phloem parenchyma cells through intervening layers of bundle sheath and mestome sheath cells (Kuo et al., 1974). Next, SWEET proteins, with 59 putative members identified in the wheat genome (Gao et al., 2018), facilitate the efflux of sucrose from the phloem parenchyma cells into the apoplasmic space (Chen et al., 2010, 2012) prior to being imported into the CCs by Sucrose/H^+^ symporters encoded by *TaSUT1* (Aoki et al., 2002). The proton motive force (pmf) required for this symport is provided by plasma membrane (PM)-localized P-type ATPases (DeWitt and Sussman, 1995), the ATP for whose operation is expected to be supplied by the oxidation of some of the symported Suc hydrolyzed via the PPi-dependent Sucrose Synthase (SUS) pathway (Geigenberger et al., 1993; Nolte and Koch, 1993; Lerchl et al., 1995; Gaxiola et al., 2012, 2016b; Khadilkar et al., 2015; Pizzio et al., 2015), where source of PPi has been proposed to be a PM-localized type I H^+^-PPase functioning as a PPi Synthase (Gaxiola et al., 2012; Pizzio et al., 2015; Scholz-Starke et al., 2019). It is worth emphasizing that there is no report in the literature providing data regarding the type I H^+^-PPase localization in wheat.

Type I H^+^-PPases are found to be ubiquitously expressed, with highest levels observed in the collection, transport, and unloading phloem as well as sink tissues (Mitsuda et al., 2001; Li et al., 2005; Paez-Valencia et al., 2011; Segami et al., 2014; Khadilkar et al., 2015; Kriegel et al., 2015; Pizzio et al., 2015; Regmi et al., 2015). In active sink tissues, the type I H^+^-PPases at the tonoplast are hypothesized to hydrolyze PPi – a byproduct of numerous anabolic reactions – to drive these biosynthetic reactions forward, while simultaneously using the free energy of PPi hydrolysis to energize the rapidly expanding vacuoles (Shiratake et al., 1997; Maeshima, 2000; Heinonen, 2001; Viotti et al., 2013; Regmi et al., 2015). Increased abundance and or activity in these tissues is expected to have a positive effect in sink strength by favoring biosynthetic reactions (Gaxiola et al., 2016b).

Under a favorable proton gradient, as is found in the phloem apoplasmic interface, a PM-localized type I H^+^-PPase (Long et al., 1995; Ratajczak et al., 1999; Langhans et al., 2001; Paez-Valencia et al., 2011; Regmi et al., 2015) has been proposed on thermodynamic (Davies et al., 1997) and structural grounds (Regmi et al., 2016), to function in reverse as a PPi-synthase (Khadilkar et al., 2015; Pizzio et al., 2015). In fact, the bidirectionality of plant membrane-bound type I H^+^-PPases has been shown \y (Scholz-Starke et al., 2019) and suggested biochemically (Façanha and de Meis, 1998; Marsh et al., 2000).

In contrast to arabidopsis, whose type I H^+^-PPase is encoded by a single copy gene, *Arabidopsis thaliana* H^+^-PPase (*AVP1*), the hexaploid wheat genome harbors three phylogenetically distinct gene paralogs encoding H^+^-PPases – *TaVP1, TaVP2*, and *TaVP3* – identified through extensive expressed sequence tag (EST) data mining and mapped to homoeologous chromosome groups 1 and 7 (Wang et al., 2009). The amino acid alignment of the consensus polypeptides encoded by these paralogs against 23 type I H^+^-PPase orthologous sequences from barley, maize, rice, sorghum, tobacco and arabidopsis showed high sequence homology, including that of a highly conserved D(X)_7_KXE motif (Wang et al., 2009). More importantly, Wang et al. (2009) used RT-PCR and dbEST surveys to examine the tissue-specific expression levels of the *TaVP*-specific transcripts and found that *TaVP3* was only expressed in developing seeds (Wang et al., 2009). *TaVP2* was found to be primarily expressed in shoot tissues, including 10-days-old seedling leaves, flag leaves, inflorescences, and uppermost internodes, but not in germinating seeds (Wang et al., 2009). *TaVP1*, which is phylogenetically more similar to *TaVP2* than *TaVP3*, was expressed more broadly, including germinating seeds, and particularly at the highest level in roots (Wang et al., 2009).

Given the versatility of type I H^+^-PPases and their utility as powerful yet simple biotechnological tools (Gaxiola et al., 2012, 2016a; Schilling et al., 2017, and references therein), we constitutively expressed *AVP1* under the control of a maize *Ubiquitin* (*UBI*) promoter in the spring wheat cultivar Bobwhite L., to understand its role in carbon partitioning. An immunohistochemical survey of various tissues showed that the wheat H^+^-PPase orthologs – TaVPs – depicted basal expression in all tissues, with maximal localization levels in the sink tissues, and the collection, transport, and unloading phloem. Using ^14^CO_2_ labeling, it was found that vegetative wheat plants partition most of the photosynthates to heterotrophic roots during the dark period, and that the delivery into roots of *UBI:AVP1* transgenic lines was prolonged in the dark period compared to controls. Moreover, during the reproductive phase, ^14^CO_2_ labeling of the terminal source flag leaves showed that the transgenic lines accumulated more ^14^C signal in the kernels. We found that TaVPs were localized in vacuoles of the scutellar epithelial cells and at the plasma membrane of SE-CC complexes using immunogold labeling. The implications of this vacuolar localization are discussed. Overall, we show evidence consistent with the expression of AVP1 augmenting carbon partitioning from source to sinks in wheat plants by either empowering the flux of carbon via phloem loading and transport, or by strengthening the sinks, which ultimately translates into improved yield.

## Materials and Methods

### Generation of Transgenic Wheat Expressing *AVP1*

The coding sequence of *AVP1* (At1g15690) was amplified from the *Arabidopsis thaliana* ecotype Col-0 cDNA and ligated into a *pENTR/D-TOPO* (Invitrogen, Carlsbad, CA, United States) entry vector. The cloned insert verified by sequencing and then subcloned into the *pMDC32* plant transformation vector (Curtis and Grossniklaus, 2003) using the Gateway LR recombination reaction (Invitrogen, Carlsbad, CA, United States). Transgenic wheat cv. Bob white expressing *pUBI:AtAVP1* was developed by biolistic transformation (Sanford et al., 1995; Vasil and Vasil, 2006). Wheat calli were developed from immature embryos (1.0–1.5 mm in length, semitransparent) and were used for bombardment. Microprojectile bombardment was performed using the Biolistic PDS-1000/He Particle Delivery System (Bio-Rad, Hercules, CA, United States). Genes of interest and selectable markers are present as intact plasmids or DNA fragments and used to coat the microprojectiles prior to shooting (Kovalchuk et al., 2009). A total of 29 independent transgenic calli were initially transformed, from which 11 independent lines were regenerated. Regenerated plants were transplanted into soil and grown under normal greenhouse conditions using standard agronomic practices to maturity. Two independent lines which showed one transgene copy number and a stable phenotype were characterized further. From the above process, an AVP1 Null line was selected as a control. AVP1 transgene copy number (Supplementary Figure S1C) and expression level were determined by qPCR (Supplementary Figure S1D).

### Total Protein Extraction

Approximately 300 mg of wheat leaves were immediately ground into a fine powder with liquid N_2_ and transferred in approximately 200 μL aliquots to screw-cap Eppendorf tubes. To each tube, 1 mL of 10% (v/v) trichloroacetic acid in −20°C acetone was added, and proteins were allowed to precipitate overnight at −20°C. The samples were centrifuged at 10000 *g* for 30 min at 4°C, followed by removal of the supernatant. The pellet was washed with −20°C acetone containing 0⋅07% β-mercaptoethanol, vortexed and centrifuged at 10000 *g* for 10 min at 4°C. The washing, vortexing, and centrifugation steps were repeated four more times. After the final centrifugation step, the supernatant was removed and the pellet dried in a tabletop vacuum for approximately 30 min. Total protein was solubilized by adding Laemmli’s buffer to the pellet. Half of the samples were boiled in a water bath for better mobility and separation of membrane proteins on SDS–PAGE. Prior to storage at −80°C, the mixture was vortexed, and centrifuged at 5000 *g* for 5 min at 4°C three times.

### Western Blot

The solubilized protein extracts from wheat leaves were then run on a 10% SDS–polyacrylamide gel, transferred to a PVDF membrane, blocked with 5% (w/v) non-fat dry milk in Tris–buffered saline with 0⋅01% Tween-20 (TBST) and probed with 1:1000 dilution of polyclonal sera generated against H^+^-PPase specific CTKAADVGADLVGKIE motif overnight at 4°C. After washing in TBST, the membrane was developed using a Bio-rad Alkaline Phosphatase Immun-Blot^®^ Colorimetric Assay kit (Bio-Rad Inc., United States^2^) according to the manufacturer’s instructions.

### Genotyping and Expression Profiling of Transgenic Lines

Plants were genotyped by PCR to confirm the presence/absence of *AVP1*. Genomic DNA was isolated using CTAB (cetyl trimethylammonium bromide) method (Porebski et al., 1997). Oligos were designed from the open reading frame and used in PCR reaction (Supplementary Table S1). The PCR cycling condition is followed by initial denaturation at 98°C–5 min (1 cycle), denaturation at 98°C–5 s, annealing at 65°C–30 s & extension at 72°C–1 min (a total of 34 cycle), and final extension at 72°C–10 min (1 cycle). PCR positive lines were identified after running 1% agarose gel (Supplementary Figure S1A).

Total RNA was extracted from leaf tissue using TRIzol reagent (Invitrogen) and a Direct-zol RNA MiniPrep Kit (Zymo Research, CA, United States) with an on-column DNase treatment. A 2.0 μg aliquot of purified RNA was used for cDNA synthesis using a High-Capacity cDNA Reverse Transcription Kit (Applied Biosystems, Victoria, Australia) and random oligo primers supplied in the kit (Supplementary Figure S1B). The expression of *AVP1*, as well as the control gene *TaGAPDH* (EU022331), were determined using Quantitative real-time PCR with gene specific primers (Supplementary Table S1). Quantitative real-time PCR was performed with KAPA SYBR^®^ Fast qRT-PCR kit Master Mix (Kapa Biosystems, Wilmington, United States) and amplification was monitored in real-time on a QuantStudioTM 6 Flex Real-Time PCR System (Applied Biosystems, Foster City, United States). Reference gene stability was assessed with the geNorm function of qBASE + software using default settings (Hellemans et al., 2007). Gene expression relative to the control gene was calculated (Supplementary Figures S1 C,D) using equation from Hellemans et al. (2007). Values are means of 3–5 biological replicates and 3 technical replicates.

### Growth Chamber Conditions

Seeds of *AVP1 Null*, *AVP1-1* and *AVP1-2* were soaked in 30% (v/v) commercial bleach with 0.01% Tween-20 for 5 min, washed several times with water, imbibed in water for 8 h, sown on moist filter paper pads in petri dishes, and incubated at room temperature in the dark for 2 days for germination. The germinated seeds were directly sown on Sunshine^®^ potting mix in pots or on Turface^®^ artificial soil in 50mL falcon tubes and grown in growth room under 12: 12: light: dark regime. The composition of the liquid fertilizer used to nourish the plants was obtained from Shavrukov et al. (2012). Photoperiod of the chamber was set up as 12 h light/12 h dark at 25°C (EGC C6 Environmental Growth Chambers), providing the light of intensity 80 μmol m^–2^ s^–1^ photosynthetic photon flux.

### Light Microscopy and Immunohistochemistry

Source leaves, leaf sheaths, flag leaves, crown roots, peduncle, spikelets, and basal rachis were excised from *AVP1 Null*, *AVP1-1*, and *AVP1-2* plants. The tissues excised processed for paraplast embedding, sectioning, and immunolabeling with anti-AVP1 rabbit polyclonal antibodies according to Regmi et al. (2015). For semi-quantitative immunohistochemistry on source leaves, the 3-3′-Diaminobenzidine development time was kept at 30s for both control and transgenic lines. Images were acquired with either a Zeiss Axioskop or Nikon Eclipe E600 (Keck Bioimaging Center, ASU) light microscope.

### High-Pressure Freezing, Immunogold Labeling, and Transmission Electron Microscopy

*Arabidopsis thaliana* H^+^-PPase *Null* seeds were germinated for 2 days under sterile conditions, dissected under 150 mM sucrose to expose the scutellar epidermis, and along with *AVP1 Null* source leaves were high-pressure frozen, freeze-substituted, embedded in LR White, ultrathin sectioned, immunogold labeled with anti-AVP1 polyclonal antibody, and imaged according to Regmi et al. (2015). Quantification of gold labeling comparing the number of 10 nm gold particles at either the PM of SEs or CCs, or the vacuoles of CCs from nine independent micrographs from three independently grown plants was done through *post hoc* pairwise comparisons using Tukey HSD.

### Pulse Chase Experiment With ^14^CO_2_ on 10-Days-Old Bobwhite and Transgenic Seedlings

Approximately 4 h into the photoperiod, 48 10-days-old wheat seedlings (12 per Bobwhite and each transgenic line, i.e., 3 plants per line per time point) were placed in a large transparent acrylic box under a halogen lamp emitting 80 μmol m^–2^ s^–1^ photosynthetic photon flux. A 1-h pulse of ^14^CO_2_ (80 μCi total ^14^C activity) was supplied to the box by mixing radioactive NaH^14^CO_3_ (specific activity: 56 mCi/mmol, concentration: 2.0 mCi/mL; MP Biomedicals, LLC) with 80% (v/v) lactic acid. Samples for radioactivity measurement (shoot and root) were weighed and collected in 20 ml scintillation vials contained 3 ml 80%(v/v) methanol. After incubating in methanol for 3 days, 0.5 mL commercial bleach (6.0% Sodium Hypochlorite) was added to each vial and incubated for 1 day. Next, 17.5 ml ECOLUME()^TM^ liquid scintillation cocktail (MP Biomedicals, LLC) was added to each vial to determine the ^14^C radioactivity of the solution fractions. Samples were incubated at room temperature with agitation at 50 rpm for all the incubation steps. Disintegrations per minute (DPM) was read by Beckman Multi-Purpose Scintillation Counter Ls6500 with a counting time of 2 min as described in Yadav et al. (2017). Any tissue harvesting in the dark period were performed under a green light source. The experiment was run twice independently – and 3 plants per line per time point were used. The experiment was run twice independently – and 3 plants per line per time point were used.

### Flag Leaf ^14^CO_2_ Labeling

Approximately 4 h into the photoperiod, the flag leaves from *AVP1 Null* and transgenic lines (Zadoks stage 75) from the main shoot were individually sealed in large transparent zip-lock bags under a halogen lamp emitting 80 μmol m^–2^ s^–1^ photosynthetic photon flux. 3 days later, 8 seeds from apical, central, and basal parts of the spikelet, and five disks ∼6mm from central part of flag leaf, and two 1.5 cm peduncle pieces from each half were measured for ^14^C activity in using the protocol described in Yadav et al. (2019). *n* = 5 plants for *AVP1 Null*, 4 plants per transgenic line.

### Evaluating Grain Set Index

The distribution of floret fertility within each spike of the main culm was mapped from the apex of the spike to the base, according to Rawson et al. (1996). Spikes were harvested during the milk stage. Established CMU (Chiang Mai University) (Rerkasem et al., 1989) and the LAC (Lumle Agricultural Research Centre) methods (Sthapit, 1988) were used to calculate the grain set index for the assessment of spike fertility:

CMU GSI (%) = (C/20) × 100, where C is the number of grains per primary and secondary floret of ten central spikelet. *n* = 3 plants per line.

LAC GSI (%) = B × 100/A, where A is the number of competent florets per spike, and B is the number of grains per spike. *n* = 4 plants per line.

The spikelets were analyzed as diagrammed in Supplementary Figure S2 C. Naming of florets within the spikelets followed (González et al., 2003).

### Evaluating Wheat Seed Germination and Post-germinative Growth

Three wheat lines (*AVP1 Null*, *AVP1*-*1*, and *AVP1-2*) were evaluated from seed germination until early seedling stage (96 h). Seeds were surface sterilized, imbibed in sterile water for 24 h in the dark, and 20 seeds per line were placed in petri dishes in the dark for 24 h. After this period, three seedlings per line were dissected to measure total fresh weight and fresh weights of coleoptile and root at 48, 72, 80, and 96 h after hydration, in addition to imaging 2 seedlings per line with a Nikon D5100 camera. At 80 h, root hairs on seminal roots were imaged using an Olympus SZX7 stereo microscope.

### Evaluation of Transgenic Wheat Under Greenhouse Conditions

Evenly sized seeds of transgenic and null segregant plants were imbibed in reverse osmosis (RO) water at room temperature for 4 h. Seeds were then placed in the dark at 4 °C for 3 days prior to transplanting into pots (diameter 150 mm and height of 150 mm). Pots were filled with a 2.0 L of cocopeat soil (South Australian Research and Development Institute (SARDI), Adelaide, Australia). Plants were grown in The Plant Accelerator (Australian Plant Phenomics Facility, University of Adelaide, Australia, latitude: −34.971353 & longitude: 138.639933) using standard agronomic practices for water management, as well as fertilizer and disease treatments. The plants were grown under natural light conditions, in a temperature-controlled greenhouse with a 22 °C daytime and 15 °C night temperatures. Measurements of plant yield data including, grain yield per plant, number of seeds per plant, head number and thousand-grain weight were taken at the time of harvesting. Phenotypic data was statistically analyzed using a one-way ANOVA and Dunnett’s test in PRISM 7 for Windows ver. 7.00 (Graphpad Software Inc., CA, United States) to determine means that were significantly different at a probability level of *P* ≤ 0.05.

### Evaluation of Transgenic Wheat Under Field Conditions

*Arabidopsis thaliana* H^+^-PPase transgenic wheat lines were grown under field trials at Glenthorne Farm, South Australia (Lat −35.056933°, Long 135.556251°). Two independent transformation events expressing *AVP1* (*AVP1*-*1*, and *AVP1-2* T_5_ generation) and AVP1 null segregates were sown. Plants were sown at a depth of 3 cm in 1 m × 0.7 M plots. Transgenic lines were sown in the middle two rows of the plot, with the outer plot rows a non-transgenic barley, cv Fielder, buffer to reduce edge effects and to stop pollen flow to neighboring plots. The barley cultivar Fielder was sown across the top and bottom of the plot (20 cm in width) to mitigate edge effects and to reduce pollen flow between the plots. An 8 cm gap was left between each plot, with a 50 cm gap left between every 5th plot to allow access. Plots were sown in a randomized block design and bird-netting placed over the trial to avoid damaged caused by birds.

Planting took place on the 25th of May 2017. High Phosophate Super Phosphate (RICHGRO, Jandakot, Western Australia, Australia) and Soluble Nitrogen Urea (RICHGRO) were applied at germination (2 weeks after sowing) at a rate of 50g m^2^ and 20g m^2^, respectively. Snail bait (Blitzem Snail & Slug Pellets, Yates, Auckland, New Zealand) was also applied at a rate of 5 g m^2^. Irrigation was supplied by rainfall, with 435 mm of rain in the growing season. At 4 weeks, establishment counts were taken of the plots. The development of the plants was observed weekly until grain harvest. Harvesting of the plant material took place on the 11th and 12th of December 2017. At harvest, total plant biomass, tiller number, the grain number, head number and grain yield per plant were recorded. *n* = 15 independent plants per line.

### Root Morphology Analysis of Transgenic Wheat Lines

Wheat transgenic lines, *AVP1* and null plants were grown in 2.5 L standard pots (150mm diameter, 190mm height) contains 2.0L Profile Porous Ceramic (PPC) Greens Grade soil (Profile Products LLC, Buffalo Grove, IL, United States) with 10 g of Osmocote (Scotts Australia Pty Ltd., Bella Vista, NSW, Australia) and watered with aqueous liquid fertilizer (1 g/L) (FertPro Australia Pty Ltd., Dinmore, QLD, Australia) every 10 days after germination. The transgenic and null wheat lines were sampled at either 3 or 6 weeks after sowing for root morphology analysis or for root biomass, respectively. Scanning of root material was found to accurately measure root length up to 3 weeks of growth (Data not shown). At three weeks, the soil was carefully removed from the plant’s root and root diameter, length, number, surface area and volume determined using a flatbed scanner (Epson, EU-88, Suwa, Japan) and RootGraph image analysis system (Cai et al., 2015). The imaging parameters used were as follows: image resolution: 600 dpi; image type: gray level; width (cm): 30; length (cm): 40 (Melino et al., 2015). After 6 weeks, roots were removed from the soil by gentle washing, dried in an oven at 80°C for 72 h, and dry weight determined. *n* = 5 independent plants per line.

## Results

### Immunohistochemical Localization of H^+^-PPases in Various Wheat Tissues

**FIGURE 1:**
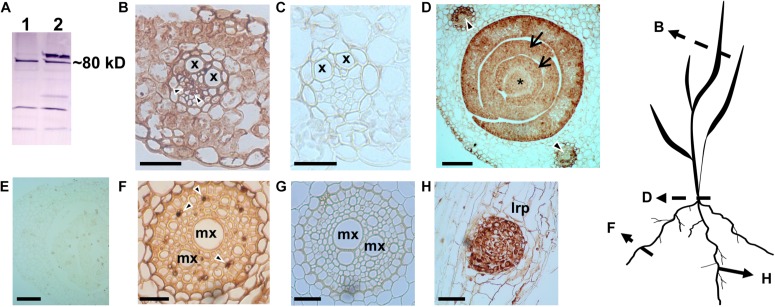
Immunohistochemical localization of wheat H^+^-PPase orthologs TaVPs in vascular and sink tissues of seedlings. **(A)** Western blot of total protein extracts from *AVP1 Null* seedlings. Lanes 1 and 2 correspond to boiled and non-boiled samples, respectively. **(B)** Transverse section of a source leaf vasculature shows a distinct immunohistochemical reaction in the phloem (arrowheads). **(C)** Pre-immune control of panel **(B)**. **(D)** Transverse section of an *AVP1 Null* seedling shows a distinct immunohistochemical reaction in the shoot apical meristem (*), leaf primordia (arrows), and vascular tissues of mature leaves (arrowheads). **(E)** Pre-immune control of panel **(D)**. **(F)** Transverse section through a root shows immunohistochemical reaction at the phloem cells (arrowheads). **(G)** Pre-immune control of panel **(F)**. **(H)** Immunohistochemical reaction at a lateral root primordium. Scale bars: **(B,C)** 50 μm; **(D,E)** 100 μm; **(F,G)** 25 μm; **(H)** 10 μm. x, xylem; mx, metaxylem; lrp, lateral root primordium. Silhouette diagram of a wheat seedling showing localization of the different sections.

In both eudicot arabidopsis and monocot rice, H^+^-PPases are basally expressed in all tissues, but also localized at both the sink tissues like root and shoot apical meristems (RAM and SAM, respectively), leaf primordia, and the phloem in source leaves (Li et al., 2005; Regmi et al., 2015). In view of this, we conducted a comparative study to examine the tissue-specific localization pattern of the TaVPs (*Triticum aestivum* vacuolar H^+^-PPases) in various wheat tissues. Previously characterized antiserum (Li et al., 2005; Park et al., 2005) generated against the highly conserved CTKAADVGADLVGKIE motif (Rea et al., 1992) in H^+^-PPases was used in this experiment, that showed a distinct band at the expected size (∼80 kDa) when immunoblotted against the total protein extracted from wheat seedlings (Figure 1A). Of note, higher molecular weight protein aggregates are recognized by the antibody if samples are not boiled. Consistent with previous outcomes (Li et al., 2005; Regmi et al., 2015), we found that TaVPs were localized in either the actively growing sink tissues or in the vasculature (Figure 1). Specifically, active sink tissues of wheat such as the SAM (Figure 1D, asterisk), leaf primordia (Figure 1D, arrows), lateral root primordium (Figure 1H), displayed the strongest signal. The TaVPs were also prominently localized in the phloem cells of the vascular bundles of the leaf sheath of a growing seedling (Figure 1D, arrowheads), in the vascular tissues of source leaves (Figure 1B, arrowheads), and in vascular elements of the stele (Figure 1F, arrowheads). Representative negative controls using pre-immune serum (Figures 1C,E,G) showed that the immunohistochemical signals observed in various wheat tissues were TaVP-specific.

### H^+^-PPases and Apoplasmic Phloem Loading in Wheat Source Leaves

**FIGURE 2:**
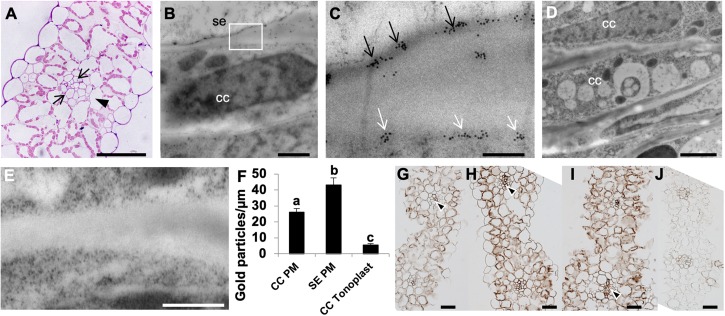
Apoplasmic phloem loading in wheat source leaves, and ultrastructural localization of H^+^-PPases. **(A)** A toluidine blue stained 500 nm semi-thin transverse section of wheat source leaf minor vein showing a layer of bundle sheath cell (arrowhead) surrounding two layers of non-photosynthetic mestome sheath cells (arrows). **(B)** Representative electron micrograph showing immunogold labeling of a longitudinal ultrathin section through a sieve element companion cell (SE-CC) complex in high-pressure frozen wheat source leaf. **(C)** Boxed inset from the panel (**B**; white box) showing distinct plasma membrane localization of anti-rabbit IgG conjugated to 10 nm gold particles in both companion cells (CC) and sieve elements (SE) (arrows). **(D,E)** Pre-immune serum negative control showing a lack of immunogold labeling. **(F)** Quantification of gold labeling comparing the number of 10 nm gold particles at either the PM of SEs or CCs, or the vacuoles of CCs from nine independent micrographs from three independently grown plants. Statistically significant differences were found in the distribution of TPV-specific gold label per μm of SE PM, CC PM and CC V (*F*_2_,_75_ = 51.12; *P* < 0⋅0001). It was inferred from *post hoc* pairwise comparisons using Tukey HSD that the mean number of gold particles per μm of SE PM (mean = 43.19, s.e. = 4.31) was significantly different from the mean number of gold particles per μm of CC PM (mean = 26.14, s.e. = 2.25), and the mean number of gold particles per μm of CC PM was also significantly different from the number of gold particles per μm of CC V (mean = 5.54, s.e. = 0.82). **(G–J)** Immunolocalization of H^+^-PPases in the source leaves of *AVP1 Null* **(G)**, *AVP1.1* **(H)**, *AVP1.2* **(I)**, and pre-immune serum negative control **(J)**. Distinct reaction in collection phloem is indicated by arrowheads. Scale bars: **(A)** 100 μm, **(B)** 10 μm, **(C)** 1 μm, **(D)** 5 μm; **(E)** 50 0nm, and (**G–J)** 50 μm.

In monocot species like wheat, phloem loading occurs in the minor and intermediate veins; whereas transport out of the leaf occurs in the large lateral veins. Transfer of the photoassimilate from the collection phloem to the transport phloem within the large veins occurs via the transverse veins that connect adjacent parallel veins (Kuo et al., 1972). In wheat source leaf minor veins, a photosynthetic bundle sheath layer (arrowhead; Figure 2A) concentrically surrounds 2 – 3 layers of non-photosynthetic mestome sheath cells (arrows; Figure 2A) as described previously (Kuo et al., 1974). It has been shown that the fluorescent tracer dye (5)6-carboxyfluorescein diacetate (CFDA) trafficked exclusively via the parallel vascular strands of wheat source leaves (Aoki et al., 2002). Furthermore, [U^14^C]-sucrose infiltrated strips of wheat source leaves showed that the radiolabel was predominantly localized in the parallel veins of the leaf (Yadav et al., 2019), as expected from apoplasmically phloem loading species. Based on previous results (Paez-Valencia et al., 2011; Regmi et al., 2015), and in the positive H^+^-PPase immunohistochemical reaction shown in vascular tissues (Figures 1B,D,F) we proceeded to document the actual membrane of H^+^-PPase residence in wheat vasculature. We used immunogold labeling in high-pressure frozen Bobwhite source leaf samples and found that the TaVPs are localized at the PM in the sieve element companion cell (SE-CC) complexes (Figures 2B,C), while negative controls performed with the pre-immune serum showed that the observed immunogold labeling was TaVP-specific (Figures 2D,E). Of note, quantification data comparing tonoplast vs. PM gold particles from nine independent micrographs showed that the distribution of TaVPs was preferentially at the PM of SEs and CCs (Figure 2F).

The transgenic wheat lines generated by transforming immature wheat embryos with *pUBI:AVP1* construct were verified for expression of *AVP1* with Q-PCR (Supplementary Figure S1D) and genotyped with specific primers (Supplementary Figures S1A,B and Supplementary Table S1). We further proceeded to ascertain that the *pUBI:AVP1* transgenic wheat lines had higher levels of H^+^-PPase in the source leaves, and upon immunolocalization and development of the 3,3′-diaminonezidine (DAB) signal for 17 s, found that the *AVP1.1* and *AVP1.2* transgenic lines had visibly stronger signal in both mesophyll cells (Figures 2H,I) and vasculature (black arrowheads, Figures 2H,I) than the control *AVP1 Null* leaf sections (Figure 2G). The pre-immune serum (Figure 2J) showed that the immunohistochemical signals observed were TaVP-specific.

### Enhanced Photosynthate Partitioning to the Heterotrophic Roots in *AVP1* Expressing Vegetative Wheat Plants

**FIGURE 3:**
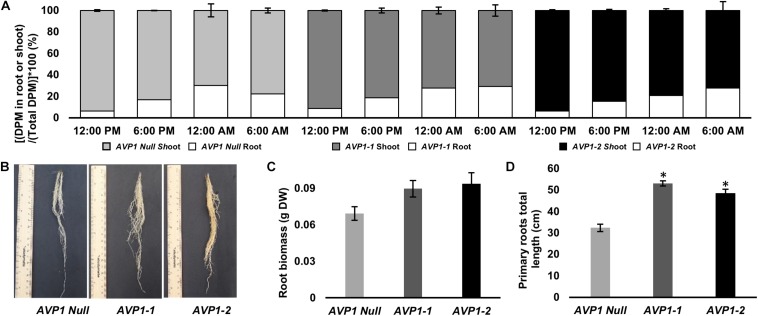
Carbon partitioning between the photoautotrophic shoot and heterotrophic root of 10-days old wheat seedlings. **(A)** Long-distance transport from the shoot to root in *AVP1 Null, AVP1-1* and *AVP1-2* plants using ^14^CO2 over light and dark periods. *n* = 3 plants per time point, per experiment, results from two independent experiments; error bars represent standard error of the mean. DPM, Disintegrations per minute. **(B)** Root growth of transgenic wheat plants expressing AVP1 under optimal growth conditions at 6 weeks after sowing. **(C)** Root biomass at 3 weeks after sowing. **(D)** Primary roots total length at 3 weeks after sowing. Plants from two independent AVP1 transformation events were grown in an inorganic kiln-fired porus ceramic matrix for three weeks prior to their root system being imaged using an Epson flatbed scanner before root mass was dried at 80°C oven for 4 days and the weight recorded. Results are the mean ± SE (*n* = 5 independent plants). Significant differences between transgenic and null plants using Dunnett’s multiple comparison test: *P < 0.05.

To monitor reduced carbon transport from source leaves into sink organs in the vegetative stages of wheat plants, we ^14^CO_2_ labeled 10-days old *AVP1 Null* seedlings (i.e., Zadoks scale 13 developmental stage). It has been suggested before that plant mobilize transitory starch in the shoots to heterotrophic roots during the dark period (Gibon et al., 2004; Smith and Stitt, 2007). 10-day old seedlings were labeled at 10:00 AM for 60 min with ^14^CO_2_, and ^14^C transport into heterotrophic roots was measured by scintillation counting at 12:00 PM (mid-day; peak of light period), 6:00 PM (onset of dark period), 12:00 AM (peak of dark period), and 6:00 AM (onset of light period) time points. As shown in Figure 3A, there is an increase in radiolabel in the heterotrophic roots during the dark period with a concomitant decrease in radiolabel in the shoots, suggesting that the majority of photosynthate flux from source to sink in vegetative wheat plants occurs during the night. Upon comparing, radiolabel levels in the roots of *AVP1 Null* and transgenic lines (Figure 3A), it is noteworthy that the accumulation of photosynthates in *AVP1 Null* roots reaches a peak between 6 PM and 12 AM before decreasing between 12 AM and 6 AM. In contrast, both transgenic lines continued to accumulate more label throughout the dark period (i.e., between 6 PM – 6 AM; Figure 3A), despite the fact that the shoot, root, and total fresh weight of *AVP1 Null*, *AVP1* transgenic seedlings were not statistically significant at the 95% confidence interval (Table 1). Of note, monitoring root growth of *AVP1 Null* and *AVP1* transgenic plants grown under optimal conditions in greenhouse indicated that *AVP1* transgenic lines developed statistically significant longer roots (45–75%) (Figure 3D). Furthermore, a positive tendency, not statistically significant, regarding root biomass (30−35% higher) in both transgenic lines when compared to *AVP1 Nulls* is evident (Figures 3B,C).

**TABLE 1:** Shoot and root biomass (fresh weight) of 10-days old *AVP1 Null* and overexpressing transgenic lines (Zadoks scale 13) used for ^14^CO_2_ labeling.

	AVP1 Null	AVP1-1	AVP1-2
**Shoot biomass (mg)**	233.67 (±8.97)	257.15 (±11.85)	235.70 (±8.84)
**Root biomass (mg)**	130.80 (±9.88)	157.12 (±11.23)	136.86 (±11.16)
**Total biomass (mg)**	364.48 (±18.43)	414.27 (±21.93)	372.56 (±19.35)

*Standard error of the mean is reported in parentheses; n = 24 plants per line.*

### ^14^CO_2_ Label Supplied From the Source Flag Leaf Into Milk Stage Kernels

While roots and young leaves are the primary sinks during the vegetative stage of a plant’s life cycle, the reproductive organs are the major sinks as plants enter the reproductive phase. During grain filling in C3 cereals like wheat, the terminal flag leaf has long been considered the primary source of photoassimilates to the kernels (Evans and Rawson, 1970; Evans, 1975; Araus and Tapia, 1987). We tested if *AVP1* expressing lines would show increased accumulation of photosynthates supplied from the flag leaf into kernels.

**FIGURE 4:**
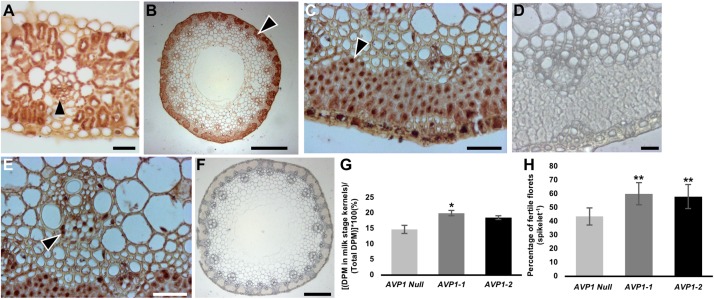
Transport of photoassimilates from flag leaves to filling stage grains in wheat plants. **(A)** Immunohistochemical localization of H^+^-PPases in transverse section of *AVP1 Null* flag leaf. Arrow head highlight immunohistochemical reaction in a minor vein. **(B–F)** Transverse section through an *AVP1 Null* peduncle shows distinct brown 3,3′-diaminobenzidine precipitate at the sub-epidermal storage parenchyma cells, arrowhead **(B–C)**, and phloem cells in vascular tissues (**E**; arrowhead). **(D,F)** Pre-immune serum negative controls of peduncle transverse sections. Scale bars: **(A)** 200 μm, **(B)** 200 μm, **(C,E)** 25 μm, **(D)** 25 μm, and **(F)** 200 μm. **(G)** Transport of ^14^C-label into milk-stage kernels after ^14^CO_2_ labeling of the flag leaves of *AVP1 Null, AVP1-1* and *AVP1-2*. *n* = 5 plants for *AVP1 Null*, 4 plants per transgenic line. Error bars represent standard error of the mean. Significant differences from the *AVP1 Null* are based on Student’s *t*-test. * indicates *P* < 0.05. **(H)** Quantification of floret fertility within each spike of the main culm of *AVP1 Null* and transgenic lines by the LAC grain set index method. *n* = 4 plants per line. Error bars represent standard error of the mean. Significant differences from the AVP1 Null are based on Student’s *t*-test. ** indicates *P* < 0.01. LAC, Lumle Agricultural Research Centre.

To further document H^+^-PPase presence in flag leaf and reproductive tissues we immunohistochemically localized TaVPs in the flag leaf and the peduncle of *AVP1 Null* plants, at Zadoks stage 59 (Figures 4A–F). Transverse sections showed clear localization of TaVPs in the vascular tissues of both the flag leaf and the peduncle (Figures 4A,E arrowheads) and sub-epidermal storage parenchyma cells in the peduncle (Figures 4B,C arrowheads). Negative controls performed with pre-immune serum showed that the observed signal was TaVP-specific (Figures 4D,F). We then compared whether the milk kernel sinks (at Zadoks stage 75) of *AVP1-1* and *AVP1-2* lines accumulated more ^14^C relative to *AVP1 Null* by labeling the source flag leaves with ^14^CO_2_, and quantified ^14^C-associated label accumulated into the milk kernels. Label accumulation in the milk stage kernels of *AVP1-1* and *AVP1-2* was about 36% and 26% higher than in *AVP1 Null*, respectively (Figure 4G).

We then followed *AVP1 Null*, *AVP1-1* and *AVP1-2* lines to maturity under growth chamber conditions and harvested the seeds to determine yield (i.e., seeds per plant, or grams of seeds per plant, or 1000 kernel weight). Under these conditions *AVP1-1* and *AVP1-2 lines* produced significantly more seeds per plant, 53.4 and 91.4%, respectively, than *AVP1 Null* (Table 2). Furthermore, the mean weight of total seeds per plant was about 36.4 and 109.1% higher in *AVP1-1* and *AVP1-2* lines than in *AVP1 Null*, respectively (Table 2). Of note, the 1000 kernel weight averages were similar among all the plants (Table 2).

**TABLE 2:** Seed yield parameters from AVP1 Null and transgenic lines grown in growth-chamber, as indicated. Standard error reported in parentheses; *n* = 4 plants per line.

	AVP1 Null	AVP1-1	AVP1-2
**Mean yield per plant (g)**	3.84 (±0.94)	5.24 (±0.83)*	8.03 (±0.92)*
**Total number of seeds per plant**	169.50 (±45.66)	260.00 (±41.54)*	324.50 (±41.68)*
**1000 kernel weight (g)**	23.03 (±1.61)	20.17 (±1.81)	24.94 (±1.89)

Significant differences from the AVP1 Null plants are based on Student’s t-test. * indicates P < 0.05.

Considering that yield and seed set are determined at the flowering stage with kernel abortion being a significant limiting factor, we also conducted an analysis of floret fertility among all lines using established methods. Using both, Lumle Agricultural Research Centre (LAC) and Chiang Mai University (CMU) method for quantifying grain set index (Sthapit, 1988; Rerkasem et al., 1989), it was found that the transgenic lines had significantly more fertile florets per spike than *AVP1 Null* (Figure 4H and Suppplementary Figure S2C).

### H^+^-PPases in Wheat Are Localized in the Scutellar Epithelial Cells of Germinating Wheat Seeds

**FIGURE 5:**
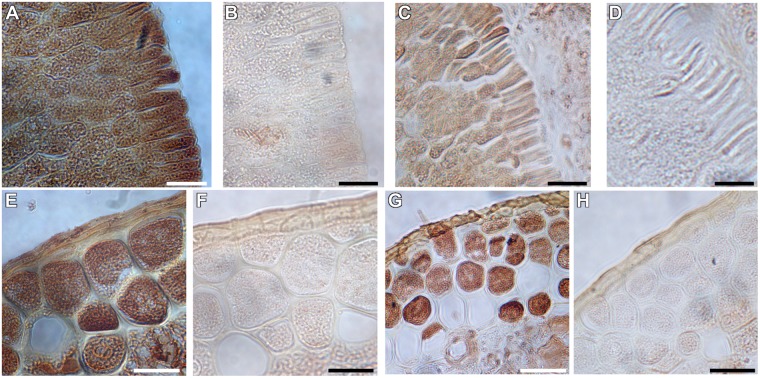
Immunohistochemical localization of H^+^-PPases and Sucrose Synthase in germinating *AVP1 Null* wheat seeds three days after imbibition. **(A,E)** Phase-contrast micrographs showing immunolocalization of H^+^-PPases in longitudinal sections of scutellar epithelial **(A)** and aleurone **(E)** cells. **(B,F)** Pre-immune control of panels **(A)** and **(E)**, respectively. **(C,G)** Phase-contrast micrographs showing distinct localization of Sucrose Synthase in the scutellar epithelial cells **(C)** and aleurone **(G)**. **(D,H)** Pre-immune control of panels **(C)** and **(G)**, respectively. Scale bars: **(A–H)** 10 μm.

Morphologically, it is apparent that the highly metabolically active scutellar epithelial cells have a single large nucleus, are highly vacuolated, and have numerous mitochondria that are distributed throughout the cytoplasm (Supplementary Figure S3). Previous studies have implicated H^+^-PPases in the germination of arabidopsis and barley seeds (Swanson and Jones, 1996; Ferjani et al., 2011). Hence, we immunohistochemically localized TaVPs in germinating wheat seeds 3 days after imbibition. H^+^-PPases were localized at the scutellar epithelial cells (Figure 5A) and in the aleurone cells (Figure 5E). Parallel negative controls with pre-immune sera (Figures 5B,F) showed that the signal in Figures 5A,E were TaVP-specific. To document if there is a possible connection between TaVP of scutellar epithelial cells and aleurone cells with the Sucrose Synthase (SUS) pathway proposed by the studies of Aoki and Perata (Perata et al., 1997; Aoki et al., 2006), we proceeded to immunolocalize SUS in the scutellar epithelial and aleurone cells. SUS orthologs were localized in both types of cells in germinating wheat seeds 3 days after imbibition (Figures 5C,G). Negative controls performed by omitting the primary antibody showed no SUS-associated signal (Figures 5D,H).

### Enhanced Post-germinative Growth in AVP1 Expressing Wheat

**FIGURE 6:**
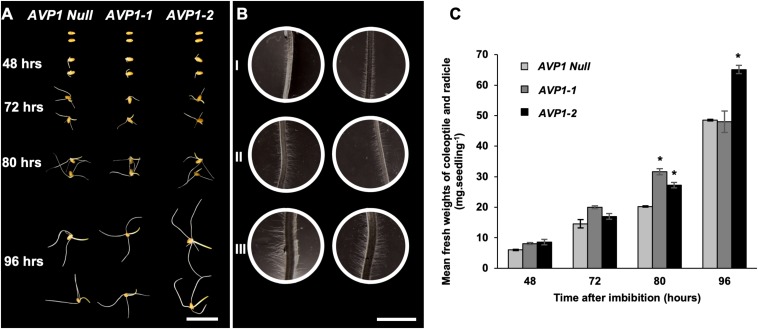
Germination of seeds and post-germinative growth of wheat seedlings in the dark. **(A)** Representative images of wheat seeds and seedlings from *AVP1 Null* and transgenic lines (*AVP1-1* and *AVP1-2*) imbibed and grown in the dark for 48, 72, 80, and 96 h. Scale bar: 2 cm. **(B)** Light micrographs of differentiation zones of seminal roots at 80 h of post-germinative growth (see A). I = *AVP1 Null*, II and III = *AVP1-1* and *AVP1-2*, respectively. Scale bar: 2 mm. **(C)** Comparative mean fresh weights of the coleoptiles and radicles from *AVP1 Null*, *AVP1-1* and *AVP1-2* lines grown in the dark (see panel **A**). *n* = 3 per line per time point. Error bars represent standard error of the mean. Significant differences from the *AVP1 Null* are based on Student’s *t*-test. * indicates *P* < 0.05.

The conspicuous of localization of TaVPs and SUS in the scutellar epithelial cells and the hypoxic conditions characteristic of this tissue suggest an interplay of both enzymes likely in carbohydrate catabolism (Primo et al., 2019). To test if the expression of the arabidopsis AVP1 could affect wheat seedling development, we measured the fresh weights of the embryonic shoot (coleoptile) and root (radicle) of *AVP1 Null*, *AVP1-1* and *AVP1-2* lines imbibed and grown in the dark for 48, 72, 80, and 96 h. It was found that at the first two time points (48 and 72 h) the only line that showed a positive tendency of post-germinative growth advantage was *AVP1-1* (Figures 6A,C). Interestingly, the other transgenic line, *AVP1-2*, picked up its growth pace at 80 and 96 h and obtained by the end of the experiment about 34% biomass increases compared to *AVP1 Null*. Additionally, upon closer examination of the material, it was also found that at 80 h, the root hairs of the differentiation zones of the seminal roots of all transgenic lines had visibly longer and denser root hairs compared to that of *AVP1 Null* (Figures 6B, I – III).

### Transgenic Wheat Expressing *AVP1* Has Increased Shoot Biomass and Grain Yield Under Greenhouse and Field Conditions

**FIGURE 7:**
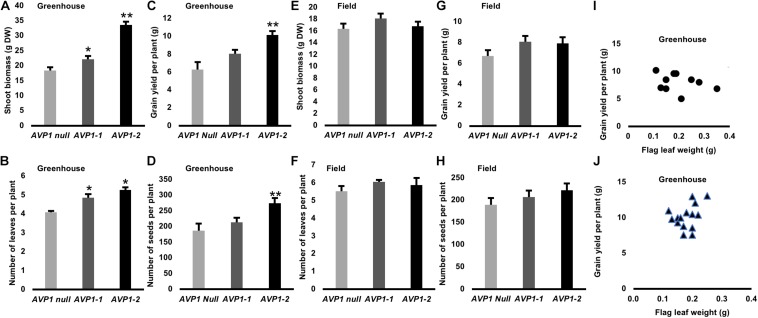
Shoot growth and yield attributes of transgenic wheat plants expressing *AVP1* under optimal growth conditions in green house and field: **(A,E)** shoot biomass (g DW), **(B,F)** Number of leaves per plant **(C,G)** Grain yield per plant, **(D,H)** Number of seeds per plant. Two independent single gene transformation events analyzed, along with the respective null segregants, which had segregated from each of the lines at the T_1_ stage. Results are the mean ± SE (*n* = 15 independent plants per line). Significant differences between transgenic and null plants using Dunnett’s multiple comparison test: *P < 0.05 and **P < 0.01. **(I)** Relationship between flag leaf weight (g) and grain yield per plant (g) of transgenic wheat plants expressing AVP1 under optimal growth conditions in green house. Two independent single gene transformation events analyzed [*AVP1-1* marked as circles, *n* = 10 independent plants **(I)** and *AVP1-2* marked as triangles, *n* = 13 independent plants **(J)**], which had segregated from each of the lines at the T_1_ stage. Person correlation coefficient (*r* = 0.3113) was calculated with two tailed *P*-values and 95% confidence interval.

Phenotypic evaluation of the transgenic wheat expressing *AVP1* and null segregants were conducted in greenhouse and field under optimal growth conditions. In greenhouse, transgenic plants expressing *AVP1* were larger (20–80%; Figure 7A), had increased leaf number (28%; Figure 7B) and had the capability to enhance grain yield per plant by 28–62% compared with null segregants (Figure 7C). *AVP1-2* lines had the greatest increase in grain yield per plant (62%), followed by *AVP1-1* (28%) lines. The enhanced grain yield in *AVP1* lines was accompanied by an increase in number of seeds per plant (10–42%; Figure 7D). The transgenic lines also flowered 7–8 days before the *AVP1* nulls (Supplementary Figure S2D).

In GM field trials, increase in shoot biomass (2–10%; Figure 7E), number of leaves (7–10%; Figure 7F) and grain yield per plant (18–20%; Figure 7G) was observed for *AVP1* expressing wheat lines compared to null segregants. Furthermore, *AVP1* transgenic lines had an increase in the number of seeds per plant (8–17%; Figure 7H). The relationship between flag leaf weight and grain yield from greenhouse grown lines was investigated (Figures 7I,J and Supplementary Figure S4), no relationship was found.

## Discussion

Maximizing the photosynthetic capacity of plants could be considered the ideal way to increase plant productivity; yet, photosynthesis is inhibited by its own products (Kottapalli et al., 2018). Strategies to circumvent this bottleneck include enhancing phloem loading/transport capacity and/or empowering the sinks (Ainsworth and Bush, 2010; Braun et al., 2013; Yadav et al., 2015). H^+^-PPases have been implicated in regulating reduced carbon transport and sink strength (Gaxiola et al., 2012, 2016b; Schilling et al., 2017). In order to test if genetic manipulation of this enzyme in wheat can improve photosynthate partitioning and yield, we expressed *AVP1* in spring wheat Bobwhite L. under the control of a maize Ubiquitin (*UBI*) promoter (Supplementary Figures S1A–D).

In this study, we first established the tissue localization pattern of H^+^-PPases encoded in the wheat genome – TaVPs – using antibody generated against the 100% conserved epitope CTKAADVGADLVGKIE (Rea et al., 1992). Higher molecular weight aggregates present in non-boiled samples suggest that H^+^-PPases may interact *in vivo* with other proteins (Figure 1A). As shown in other plants (Paez-Valencia et al., 2011; Segami et al., 2014; Regmi et al., 2015) H^+^-PPases showed basal expression in most cells and maximal localization signal in sink tissues (Figures 1D,H) and collection (Figures 1B–D, 2G–J, 4A), and transport phloem (Figures 1F, 4E).

TaVPases were localized in the vascular tissues (Figures 1B,D,F, 2G–I, 4A,E) suggesting that in wheat these enzymes could be involved in photosynthate partition (Regmi et al., 2015). Reports of symplasmic fluorescent tracer (5)6-Carboxyfluorescein diacetate (CFDA) and [U^14^C]-sucrose localized in the vascular bundles of wheat source leaves are consistent with apoplasmic loading (Figure 2C; Aoki et al., 2002; Yadav et al., 2019). Unlike actively dividing cells where H^+^-PPases are localized at the tonoplast (Maeshima and Yoshida, 1989; Maeshima, 2000; Heinonen, 2001; Regmi et al., 2015), H^+^-PPases have been shown to be localized at the plasma membrane (PM) in the sieve element companion cell (SE-CC) complexes of apoplasmic loaders like arabidopsis (Paez-Valencia et al., 2011), rice (Regmi et al., 2015), and ricinus (Long et al., 1995; Langhans et al., 2001). In agreement, TaVP-specific immunogold labeling was found to be at the PM of SE-CC complexes (Figures 2B,C).

The model postulated by Gaxiola et al. (2012) proposed that PM-localized H^+^-PPases in SE-CC complexes work as PPi synthases and favor the SUS-mediated hydrolysis of Suc. Given that the evidence pointed to wheat being an apoplasmic phloem loader and that wheat H^+^-PPase orthologs were distinctly localized at the PM of SE-CC complexes (Figures 2B,C) together with the fact that immunohistochemical reactions in the transgenic lines are consistent with augmented presence of H^+^-PPase in collection phloem (Figures 2G–I arrow heads), we tested if the constitutive expression of *AVP1* in wheat could affect carbon flux from source to sink (Gaxiola et al., 2012; Khadilkar et al., 2015). ^14^CO_2_ labeling of vegetative 10-days old *AVP1 Null* seedlings showed that more than 50% of the root ^14^C label was transported from the shoots to the roots during the dark period with peak transport attained at midnight before declining by the onset of the light period (Figure 3A). In contrast, the constitutively *AVP1* expressing lines *AVP1-1* and *AVP1-2* continued to transport ^14^C label to the roots throughout the dark period (Figure 3A). This behavior suggests that in transgenic wheat the constitutive expression of type I H^+^-PPases is instrumental in prolonging the transport of reduced carbon from source to sink. Of note, we cannot rule out that in transgenic *AVP1* plants, H^+^-PPase expression could not only influence the loading/transport of reduced carbon from the source, but also confer increased sink strength by favoring biosynthetic reactions (Gaxiola et al., 2016b).

Beyond the vegetative phase of wheat, the most important source-sink relationship occurs during the reproductive stage. The majority of photoassimilates destined for phloem unloading via the peduncle (uppermost stem internode) into developing sink kernels are synthesized in the terminal flag leaf blade (Evans and Rawson, 1970; Evans, 1975; Araus and Tapia, 1987). Upon immunohistochemical labeling, it was found that TaVPs were localized in the vascular tissues of the flag leaf and the peduncle (Figures 4A,E arrow heads). In addition to the vascular tissues, a conspicuous localization of TaVPs in the sub-epidermal storage parenchyma cells in the peduncle, known to store simple and complex carbohydrates (Scofield et al., 2009), was evident (Figures 4B,C arrow heads). Currently, we cannot provide any clear role for TaVP presence in the sub-epidermal storage parenchyma cells, and further research is needed to clarify this point.

We labeled the terminal flag leaf blades with ^14^CO_2_ and assessed whether the transgenic lines accumulated more ^14^C label in the milk stage kernels than Bobwhite. As shown in Figure 4G, kernels from *AVP1-1* and *AVP1-2* accrued 36% and 26% more ^14^C label on average compared to *AVP1 Null*. Here too, it is clear that increased abundance of H^+^-PPase augments carbon partition from source to sinks. Interestingly, *AVP1-1* and *AVP1-2* lines generated more grains per plant – 260 ± 41.5 and 324 ± 41.7, respectively – than *AVP1 Null* (169.5 ± 45.7) lines (Table 2) when grown in growth chamber. Seeds constitute the primary yield component of crops like wheat and the yield per plant is directly contingent on sink strength and sucrose supply from the source leaves (Xu et al., 2012). Therefore, it can be plausibly inferred from our data that the transgenic lines not only have increased ^14^C transport into both the roots and kernels (Figures 3B, 4G), but also presumably have stronger sinks. Interestingly, the *AVP1* expressing lines did not show any correlation between flag leaf weight and seed production (Figures 7I,J and Supplementary Figure S4). The lack of correlation further indicates that the effect of the H^+^-PPase in carbon partition is more due to its role in transport and or sink strength rather than its direct effects on photosynthesis.

Final seed set is also determined at the flowering stage with kernel abortion via programed cell death causing major losses in yield (Boyer and McLaughlin, 2006). Our finding that the *AVP1 Null* and transgenic lines had statistically similar number of spikes per plant (Supplementary Figure S2A), yet the transgenic lines outperformed the *AVP1 Null* in terms of seeds produced per plant, suggests that there is a lower rate of kernel abortion in the transgenic lines. In keeping, the quantification of grain set index showed ∼38 to 33% more fertile florets per spike in the *AVP1-1* and *AVP1-2* lines, respectively, compared to the *AVP1 Null* (Figure 4H and Supplementary Figure S2C). However, the role of H^+^-PPases in the mechanism underlying this phenotype is unclear and needs further detailed investigation.

From earlier studies, the role of H^+^-PPases was found not only to be limited to maintaining PPi homeostasis in meristematic and phloem tissues (Maeshima, 2000; Pizzio et al., 2015), but also in germinating arabidopsis seeds (Ferjani et al., 2011) and in the aleurone cells of barley seeds (Wisniewski and Rogowsky, 2004). Given the role H^+^-PPases play in sucrose partitioning between source and sinks (Li et al., 2005; Khadilkar et al., 2015; Pizzio et al., 2015), it is considered important to study their role in germinating wheat seeds (Schilling et al., 2017) where the early heterotrophic growth of the embryonic shoots (coleoptile) and roots (radicle) is driven by the endosperm starch (Edelman et al., 1959; Aoki et al., 2006). The immunolocalization data of *TaVP* and *TaSUS* in the scutellar epithelial cells (Figures 5A,C) suggests a possible connection between *TaVP* and the Sucrose Synthase (SUS) pathway proposed by the studies of Aoki and Perata (Perata et al., 1997; Aoki et al., 2006). It is possible to speculate that the H^+^-PPases could be instrumental in scavenging cytosolic pyrophosphate, thereby promoting the synthesis of sucrose via the PPi-sensitive Sucrose Synthase pathway (Ferjani et al., 2011).

We measured the fresh weights of the embryonic shoots (coleoptile) and roots (radicle) of *AVP1 Null*, *AVP1-1*, and *AVP1-2* lines, and found that over a period of 96 h of etiolated post-germinative growth, the transgenic line *AVP1-2* outperformed the *AVP1 Null* by 34% (Figures 6A,C). Interestingly, seeds from the *AVP1-1* did not attain significantly different germinative growth when compared to *AVP1 Nulls*, however, they did develop larger and denser root hairs similar to those of *AVP1-2* seedlings (Figure 6B). An enhanced post-germinative growth of wheat seedlings in the *AVP1-2* line relative to *AVP1 Nulls* might facilitate early phases of wheat seedling establishment. Given that successful seedling establishment is the first critical step for crop production (Finch-Savage and Bassel, 2015), it would be important to test whether these results from AVP1 expressing lines translate to more realistic field conditions. Additionally, upon closer examination during the same experiment, it was also found that the root hairs of the differentiation zones of the seminal roots in all *AVP1* transgenic lines were visibly longer and denser when compared to that of *AVP1 Nulls* (Figure 6B, I – III). Root hairs increase the absorptive surface area of the roots and are implicated in rhizosphere-mediated phosphorus nutrition of plants (Itoh and Barber, 1983; Schjørring and Nielsen, 1987; Föhse et al., 1991; Caradus, 1994). More pertinently, *AVP1* expression in diverse plants including arabidopsis, tomato, and rice induced enhanced phosphorus nutrition under phosphorus-limiting conditions (Yang et al., 2014). Whether this phenotype is conserved in *AVP1* expressing wheat lines merits further investigation.

The transgenic wheat lines expressing *AVP1* were evaluated in green house and field under optimal growth conditions to verify whether increased photosynthate partitioning from source flag leaf to filling grains results in improved yield or not. In this study, *AVP1* expressing wheat plants produced greater shoot biomass associated with an increase leaf number compared to null segregants (Figure 7). In addition, *AVP1* expressing wheat produced a greater root system and had a significantly higher grain yield. The enhanced grain yield in *AVP1* lines was accompanied by an increase in the number of seeds per plant. Our results are consistent with our work on barley (Schilling et al., 2014), supporting that *AVP1*-expression could lead to increase in shoot biomass and grain yield under non-saline conditions. Other studies have also shown that *AVP1* (or *AVP1* ortholog) expressing plants generated larger shoot biomass and/or yield compared to plants without this gene in non-saline conditions (Yang et al., 2007; Lv et al., 2008; Li et al., 2010; Vercruyssen et al., 2011; Gouiaa et al., 2012) but as this has not been the main focus of their work and so little commentary has been made on these results. Evidence that is more recent suggests that vacuolar H^+^-PPases have other roles, including the acquisition and partitioning of organic carbon and other nutrients between the different organs of the plant body (Gaxiola et al., 2001, 2016b; Ferjani et al., 2011; Ferjani and Maeshima, 2016; Schilling et al., 2017). Furthermore, our study suggests that the upregulation of the H^+^-PPase localized to the plasma membrane of SE–CC complexes increases the PPi supply facilitating sucrose loading and transport from source to sink tissues (Gaxiola et al., 2012, 2016b; Khadilkar et al., 2015), which possibly explain the improved grain yield of transgenic wheat expressing *AVP1*. However, we cannot rule out that the upregulation of the H^+^-PPase in sinks could also enhanced sink strength by favoring biosynthetic reactions (Gaxiola et al., 2016b). Generation of transgenic plants carrying source and sink specific AVP1 expression cassettes could help to understand the specific role of H^+^-PPase in carbon partition.

## Conclusion

As reported in evolutionarily divergent plants including physcomitrella (Regmi et al., 2017), arabidopsis (Paez-Valencia et al., 2011; Viotti et al., 2013), and rice (Regmi et al., 2015), H^+^-PPase orthologs in wheat show dual membrane localization; with distinct PM localization in the SE-CC complexes of source leaf minor veins. *AVP1* expressing wheat plants in the vegetative stage had improved photosynthate transport from source leaves to roots. These transgenic wheat lines also showed increased photosynthate partitioning from the source flag leaf to filling grains resulting in improved yield – increasing seeds produced per plant – suggesting more efficient transport and/or stronger sinks. It would be important to generate tissue specific chimeras to test if the augmented reduced carbon translocation requires H^+^-PPase expression either at the source and/or at the sinks. Interestingly, an enhanced post-germinative growth of wheat seedlings in the *AVP1-1* line relative to *AVP1 Null* was evident. This early advantage can facilitate the crucial phase of wheat seedling establishment, a key agriculturally desirable phenotype. It is shown that the expression of *AVP1* in wheat improves the grain yield when grown in green house and field conditions under optimal growing conditions. Furthermore, the enhanced yield of the plants was accompanied by increase in number of seeds, shoot biomass, tillers and enhanced root growth in both controlled and/or field environments.

## Data Availability Statement

The raw data supporting the conclusions of this article will be made available by the authors, without undue reservation, to any qualified researcher.

## Author Contributions

SR and RG conceived the research plans. KR, JGF, and LL performed most of the lab experiments. DP made the constructs. KY transformed the plants, screened for transgenic lines, and performed real-time PCR experiments and all glasshouse and field data acquisition. SS performed the root phenotyping. CT, LS, and JG designed and managed the Adelaide greenhouse and field trials. RK genotyped the plants. UY, JGF, and BA performed the radioactive sucrose infiltration assays, and helped develop the ^14^CO_2_ labeling experiments. KR and RG wrote the manuscript. All authors reviewed and approved the final version of the manuscript.

## Conflict of Interest

The authors declare that the research was conducted in the absence of any commercial or financial relationships that could be construed as a potential conflict of interest.

## Footnotes

1 http://www.fao.org/faostat/en/#data

2 www.bio-rad.com

## Supplementary Material

FIGURE S1 Molecular evaluation of *AVP1* expressing transgenic wheat plants. **(A)** Genotyping for the presence or absence of AVP1 using polymerase chain reaction (PCR) with AVP1-specific primers and TaGAPDH specific primers (internal control). **(B)** Expression analysis of AVP1 using reverse transcription PCR (RT-PCR) with AVP1-specific and TaGAPDH-specific primers (internal control) for transgenic wheat expressing AVP1 and null segregants. Lane WT is wildtype wheat plant, lanes 1, 2 and 3 (AVP1-1) & 4, 5 and 6 (AVP1-2) are transgenic wheat plants, lanes 7, 8 and 9 are null segregants and lane (–) is a negative control (water). **(C)** Transgene copy numbers in *AVP1*-transgenic wheat plants estimated by Q-PCR. **(D)** Relative expression of *AVP1* gene of transgenic wheat expressing *AVP1* compared to null segregants. Expression data displayed as normalized relative quantity (NRQ) for two independent single gene transformation events. Values are means of 3–5 biological replicates and three technical replicates. Error bars represent standard error of the mean.

FIGURE S2 Differences in reproductive tissue development between AVP1 Null and transgenic lines. **(A)** The number of spikes produced by AVP1 Null and transgenic lines. *n* = 4 plants per line. No significant differences at the 95% confidence interval were found. *AVP1-Null* = 3.00 ± 1.41; *AVP1-1* = 3.75 ± 0.50; and *AVP1-2* = 3.5 ± 0.58. **(B)** Schematic diagram illustrating spikelets’ positions within the spike as well as the florets’ positions that were evaluated in the floret fertility study. **(C)** Quantification of floret fertility within each spike of the main culm of AVP1 Null and transgenic lines by the CMU grain set index method. *n* = 3 plants per line with grains of 20 florets of the 10 central spikelets evaluated. Significant differences from the AVP1 Null are based on Student’s *t*-test.* indicates *P* < 0.05. Error bars represent standard error of the mean. **(D)** Flowering time of transgenic wheat expressing AVP1 compared to null segregants. Flowering data are the mean ± SE (*n* = 15 independent plants per line). Significant differences between transgenic and null plants using Dunnett’s multiple comparison test: **P* < 0.05 and ***P* < 0.01.

FIGURE S3 Ultrastructural morphology of high-pressure frozen wheat scutellar epithelial cells two days after imbibition. The cells have prominent nuclei (n), are elongated and highly vacuolated (v) with numerous mitochondria (m) interspersed throughout the cytoplasm.

FIGURE S4 Relationship between flag leaf weight (g) and grain yield per plant (g) of transgenic wheat plants expressing AVP1 under optimal growth conditions in green house. Null segregants (AVP1 Null marked as square, *n* = 12 independent plants), which segregated from each of the lines at the T_1_ stage. Person correlation coefficient (*r* = 0.3113) was calculated with two tailed P values and 95% confidence interval.

TABLE S1 List of primers used for genotyping and quantifying expression in *AVP1*, *AVP1-2* lines.
