# Supplementary figures and images for: Improved Yield and Photosynthate Partitioning in AVP1 Expressing Wheat (Triticum aestivum) Plants
Source: Front Plant Sci. 2020 Mar 17;11:273. doi: 10.3389/fpls.2020.00273 (PMC7090233; doi:10.3389/fpls.2020.00273)

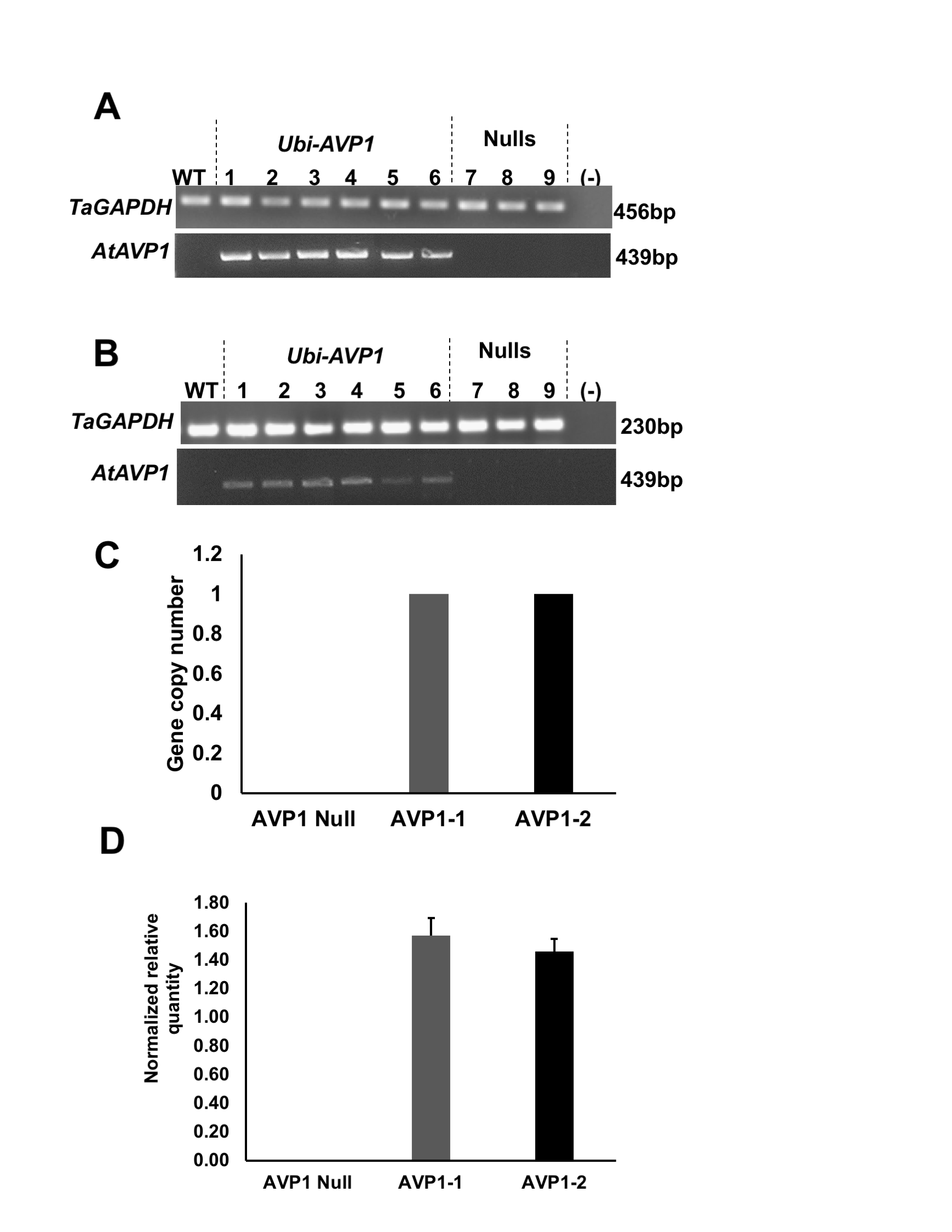

Supplement: FIGURE S1 — Molecular evaluation of AVP1 expressing transgenic wheat plants. (A) Genotyping for the presence or absence of AVP1 using polymerase chain reaction (PCR) with AVP1-specific primers and TaGAPDH specific primers (internal control). (B) Expression analysis of AVP1 using reverse transcription PCR (RT-PCR) with AVP1-specific and TaGAPDH-specific primers (internal control) for transgenic wheat expressing AVP1 and null segregants. Lane WT is wildtype wheat plant, lanes 1, 2 and 3 (AVP1-1) & 4, 5 and 6 (AVP1-2) are transgenic wheat plants, lanes 7, 8 and 9 are null segregants and lane (–) is a negative control (water). (C) Transgene copy numbers in AVP1-transgenic wheat plants estimated by Q-PCR. (D) Relative expression of AVP1 gene of transgenic wheat expressing AVP1 compared to null segregants. Expression data displayed as normalized relative quantity (NRQ) for two independent single gene transformation events. Values are means of 3–5 biological replicates and three technical replicates. Error bars represent standard error of the mean. [file Image_1.TIFF]

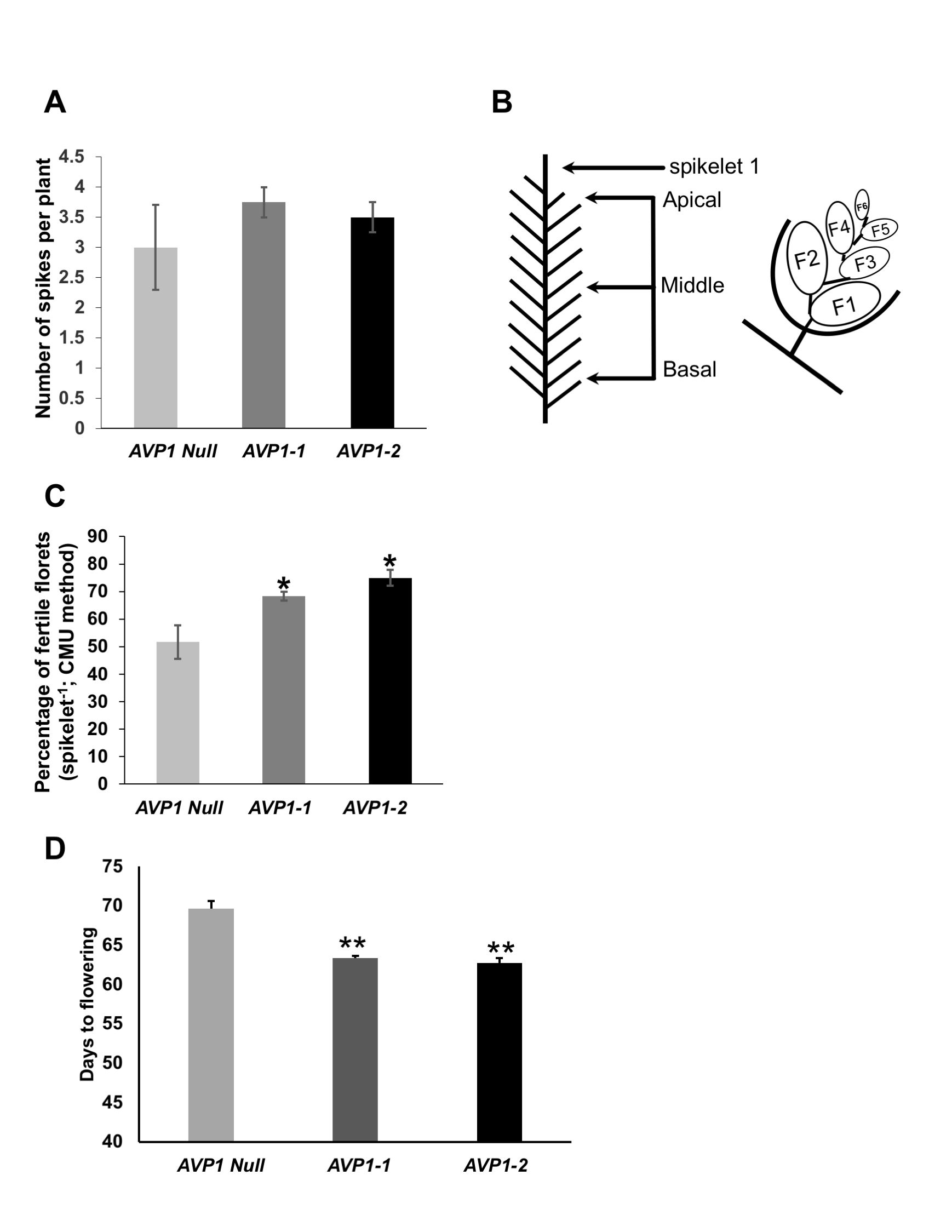

Supplement: FIGURE S2 — Differences in reproductive tissue development between AVP1 Null and transgenic lines. (A) The number of spikes produced by AVP1 Null and transgenic lines. n = 4 plants per line. No significant differences at the 95% confidence interval were found. AVP1-Null = 3.00 ± 1.41; AVP1-1 = 3.75 ± 0.50; and AVP1-2 = 3.5 ± 0.58. (B) Schematic diagram illustrating spikelets’ positions within the spike as well as the florets’ positions that were evaluated in the floret fertility study. (C) Quantification of floret fertility within each spike of the main culm of AVP1 Null and transgenic lines by the CMU grain set index method. n = 3 plants per line with grains of 20 florets of the 10 central spikelets evaluated. Significant differences from the AVP1 Null are based on Student’s t-test.* indicates P < 0.05. Error bars represent standard error of the mean. (D) Flowering time of transgenic wheat expressing AVP1 compared to null segregants. Flowering data are the mean ± SE (n = 15 independent plants per line). Significant differences between transgenic and null plants using Dunnett’s multiple comparison test: *P < 0.05 and **P < 0.01. [file Image_2.TIFF]

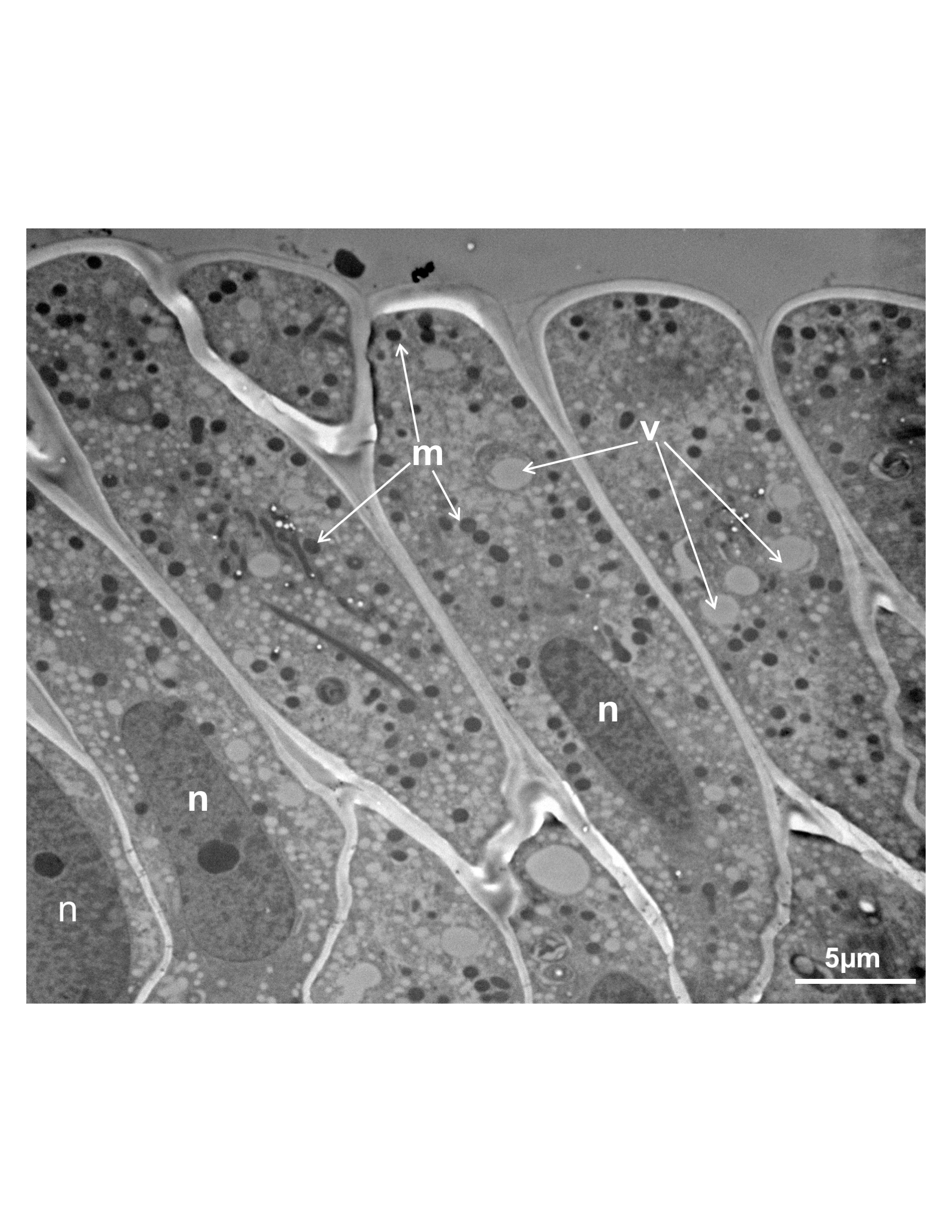

Supplement: FIGURE S3 — Ultrastructural morphology of high-pressure frozen wheat scutellar epithelial cells two days after imbibition. The cells have prominent nuclei (n), are elongated and highly vacuolated (v) with numerous mitochondria (m) interspersed throughout the cytoplasm. [file Image_3.TIFF]

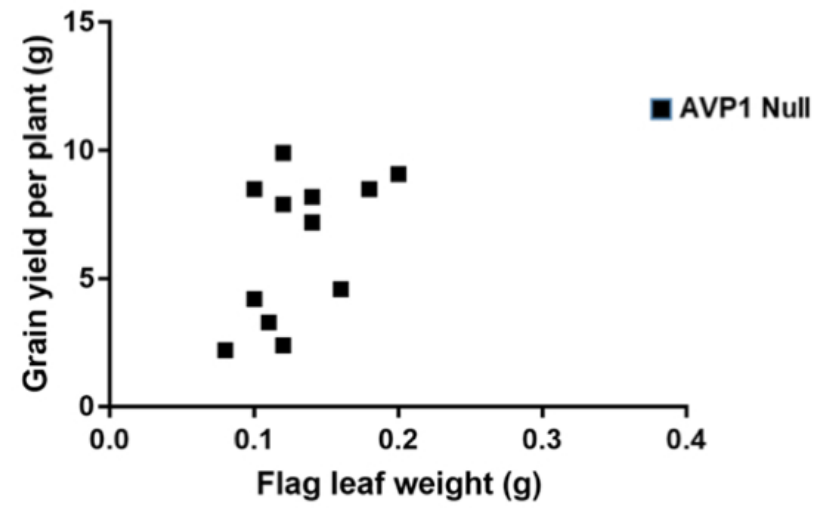

Supplement: FIGURE S4 — Relationship between flag leaf weight (g) and grain yield per plant (g) of transgenic wheat plants expressing AVP1 under optimal growth conditions in green house. Null segregants (AVP1 Null marked as square, n = 12 independent plants), which segregated from each of the lines at the T1 stage. Person correlation coefficient (r = 0.3113) was calculated with two tailed P values and 95% confidence interval. [file Table_1.pdf]
