# Supplementary material for: Improved Yield and Photosynthate Partitioning in AVP1 Expressing Wheat (Triticum aestivum) Plants
Source: Front Plant Sci. 2020 Mar 17;11:273. doi: 10.3389/fpls.2020.00273 (PMC7090233; doi:10.3389/fpls.2020.00273)
Supplement: TABLE S1 — List of primers used for genotyping and quantifying expression in AVP1, AVP1-2 lines. [file Data_Sheet_1.PDF]

Table S1: List of primers used for genotyping and quantifying expression in *AVP1-1*, *AVP1-2* lines.

		AVP1
Genotyping	Forward Primer	ATG GTG GCG CCT GCT TTG TTA CCG GAG
Genotyping	Reverse Primer	CAC AGG GCT GTA GGC GTT AC
qPCR	Forward Primer	TGT TTT GAC CCC TAA AGT TAT C
qPCR	Reverse Primer	TGG CTC TGA ACC CTT TGG TC
		TaGAPDH
qPCR	Forward Primer	TTC AAC ATC ATT CCA AGC AGC A
qPCR	Reverse Primer	CGT AAC CCA AAA TGC CCT TG
